# New Concept of Combined Microwave Delay Lines for Noise Radar-Based Remote Sensors

**DOI:** 10.3390/s19224842

**Published:** 2019-11-06

**Authors:** Zenon Szczepaniak, Waldemar Susek

**Affiliations:** Faculty of Electronics, Military University of Technology, gen. Sylwestra Kaliskiego St. No. 2, 00-908 Warsaw, Poland; zenon.szczepaniak@wat.edu.pl

**Keywords:** noise radar, radar signal processing techniques, analogue correlation, modern radar applications, delay line

## Abstract

Delay lines with a tunable length are used in a number of applications in the field of microwave techniques. The digitally-controlled analogue wideband delay line is particularly useful in noise radar applications as a precise detector of movement. In order to perform coherent reception in the noise radar, a delay line with a variable delay value is required. To address this issue, this paper comprises a new concept of a digitally-controlled delay line with a set of fine distance gates. In the paper, a solution for micro-movement detection is proposed, which is based on direct signal processing in the time domain with the use of a microwave analogue correlator. This concept assumes the use of a microwave analogue tapped delay line structure. It was found that the optimal solution for a noise radar with an analogue signal correlator is a combined delay line consisting of switched reference sections, a tapped delay line, and a precision phase shifter. The combined delay line presented in this paper is dedicated to serving as the adjustable reference delay for a noise radar intended for the detection of micro-movement. The paper contains the calculation results and delay line implementation for a given example. The new structure of the analogue tapped delay line with the calculation of optimal parameters is also presented. The precise detector of movement can be successfully used for the remote sensing of human vital signs (especially through-the-wall), e.g., breathing and heart beating, with the simultaneous determination of position.

## 1. Introduction

Delay lines with a tunable length are used in a number of applications in the field of microwave techniques [1,2,3,4]. The possible applications depend on the length of the line and generally they may be divided as follows: phase shifters, phase correctors, impedance tuning stubs, or time delay references.

The simplest form of a fixed microwave delay line is a section of a transmission line with a specified length, preferably without the effect of group velocity dispersion. In this case, the group delay *T_g_* may be expressed by

(1) $$T_{g}=\frac{L}{v_{g}},$$

where *L* is the length of the transmission line and *v_g_* is the group velocity, defined as

(2) $$v_{g}=\frac{\partial \omega }{\partial \beta }.$$

In Equation (2), variable *β* is the propagation constant and *ω* is the angular frequency. For the frequency range, where the group velocity dispersion may be neglected, the group velocity may be approximated by the expression for phase velocity of *v*_φ_ = *ω*/*β* that gives

(3) $$T_{g}=\frac{L\beta }{\omega }.$$

The time delay of a section of a transmission line is proportional to its physical length. In order to achieve large time delays, adequately long sections of the transmission line have to be used. Because the Relationship (3) also contains a phase constant and further electrical length *βL*, the time delay is a function of the parameters (especially permittivity) of the material filling of the transmission line. Therefore, there is a possibility of obtaining bigger time delays per unit length of the transmission line when it is filled with material with a high permittivity *ε_r_*. Therefore, for a TEM line, one may write

(4) $$T_{g}=\frac{\sqrt{\varepsilon_{r}}L}{c}.$$

An example of this relationship is as follows: one meter of free space propagation or a perfect TEM line with air filling corresponds to 3.33 ns of time delay. In order to have a properly working delay line set, the propagation of the delayed wave should not be affected by the group velocity dispersion. This requirement is fulfilled when TEM lines are used, for example, coaxial lines or a planar line microstrip or coplanar. The bandwidth of the transmitted signal should be limited in order to not excite an unwanted waveguide (not TEM) mode of propagation.

The digitally-tunable delay line allows a number of pre-defined values of the time delay or length corresponding to the smallest assumed delay step to be set [5]. In the case of analogue tuning of a line’s electrical length, theoretically, it is possible to obtain any value of delay from a predefined delay range.

An electronically-controlled microwave delay line is part of an analogue correlation detector used in a noise radar. The most basic design of a noise radar consists of an analogue receiver and analogue delay line with the ability to adjust the time delay. The current development of noise radars mainly concerns the use of advanced techniques of digital signal processing in order to obtain fully-digital correlation receivers [6,7,8]. However, a noise radar with an analogue correlation receiver with a tunable reference delay line may still be a useful enough solution, especially for micro-movement detection with range determination. In comparison, a typical CW (Continuous Wave) Doppler radar only detects the micro-movement speed, e.g., breathing or heartbeat, without information about the distance to the measured object [9]. Ultra WideBand (UWB) radars including noise or pseudorandom noise-based radars are used in various applications, including remote vital sign detection for rescue, security, and medical care or diagnostics [10,11].

To perform coherent reception in noise radar, a delay line with a constant or variable delay value is required. By analyzing the possibility of micro-movement detection, which is also described in the literature by the term micro-Doppler detection [12], one may notice that current investigations concern the development of algorithms to perform digital signal processing in the baseband in the frequency domain (spectrum analysis). The main issue is finding the spectrum shift of the received signal with respect to the transmitted signal. This shift may be equal to an order of MHz for a spectrum width equal to dozens of MHz. It is very difficult to detect such a small shift and new specialized methods should be developed.

The research question is to find the optimized analogue microwave tunable delay line in order to adjust the operating point of the correlation detector. In this paper, a solution for micro-movement detection is proposed, which is based on direct signal processing in the time domain with the use of a microwave analogue correlator. Therefore, to address this issue, further sections of the paper comprise a new concept of a digitally-controlled delay line with a set of fine distance gates. This concept assumes the use of a combined set of three lines, including a new version of a tapped delay line. In order to verify the concept, the example of micro-movement detection is presented, with the following assumptions: target distance 12 m, micro-movement amplitude 1 mm, noise radar with bandwidth 1 GHz, and center frequency 6.5 GHz.

The combined delay line presented in this paper is dedicated to serving as the adjustable reference delay for a noise radar intended for the detection of micro-movement. This approach allows the measurement setup to be simplified and increases the possibility of micro-movement detection. The delay line’s physical structure depends on the dedicated application of a given radar system. The source of micro-movement may be different, for example, it may be a vital activity of the human body or its organs, such as chest movement due to heartbeat and breathing.

## 2. Principle of Noise Radar Technology

Noise radars belong to radars which use random or pseudorandom signals for probing purposes and coherent detection techniques for receiving signals. Their fundamental parameters are the following: wide bandwidth, low power density, and high accuracy for distance and velocity measurements, which results from the properties of the ambiguity function of the wideband noise signal. A correlation receiver is a typical element of a noise radar. Coherent reception needs delay lines of constant or variable parameters to be applied in the receiving systems. The delay line allows one to memorize a sample of the transmitted noise signal for an amount of time delay resulting from the round-trip of the transmitted signal, from the radar transmitter to the target and back to the radar receiver. In general, the principle of the operation of a noise radar and its various structures has been widely described in the scientific literature, including the basic use of the radar, which is target range and radial velocity estimation [13,14,15,16,17,18,19,20].

The radar transmitter allows the noise signal from the frequency range, which is said to be from 1 to 18 GHz, and bandwidth of about 2 GHz, to be generated. The primary noise source in the transmitter may be realized by means of a semiconductor avalanche diode or a Zener’s diode. The signal from the transmitter is fed to a transmitting antenna with the use of a directional coupler to the delay line. The signal collected by a receiving antenna is amplified and filtered in the front-end receiver and further correlated with a copy of the transmitted signal delayed by a delay line. When the time delay value *T_DL_* is equal to the time T corresponding to the round-trip of the transmitted signal (from the radar to the target and back to the radar), a so-called “correlation peak” will appear at the output of the correlation detector. The existence of a distinguishable value of the signal *S_OUT_*(*t*) occurring for a given value of time delay *T_DL_* provided by a delay line allows target detection and distance estimation according to the formula *R* = c*T_DL_*/2.

**Figure 1:**
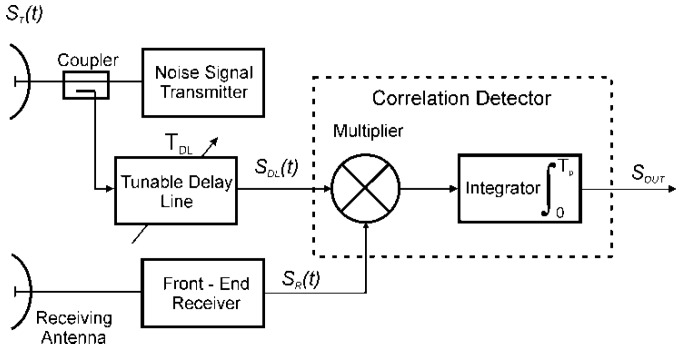
Outline of a noise radar.

For the following consideration, it is assumed that the transmitter generates a signal in the form of noise with a limited bandwidth and normal distribution, with an average value equal to zero and a variance equal to σ^2^. The signal generated in the transmitter can be described by the Expression (5), whereas signals in the individual points of the system (Figure 1) are described by Relations (6) and (7):

(5) $$S_{T}\left(t\right)=x\left(t\right)\cos\left(\omega_{0}t\right)-y\left(t\right)\sin\left(\omega_{0}t\right)$$

(6) $$S_{DL}\left(t\right)=k_{1}S_{T}\left(t-T_{DL}\right)$$

(7) $$S_{R}\left(t\right)=k_{2}S_{T}\left(t-T-\frac{2D\left(t\right)}{c}\right),$$

where *T* is the signal delay time on the radar-object-radar path, *T_DL_* is the delay time of the delay line, *D*(*t*) is the micro-movement of certain object parts, *ω*_0_ is the median frequency of the band occupied by the noise signal, *x*(*t*) and *y*(*t*) are independent realizations of the stationary random process with a Gaussian distribution having an average value equal to zero, and *k*_1_ and *k*_2_ are the propagation coefficients.

As a result of the multiplication of signals described by (6) and (7), and afterwards, integration of the obtained products, the output signal *S_OUT_*(*t*) can be expressed in the following Form (8):

(8) $$S_{OUT}\left(t\right)=A\left(\Delta T\right)\cos\left[\omega_{0}\left(\Delta T+\frac{2D\left(t\right)}{c}\right)\right],$$

where Δ*T* = *T* − *T_DL_* and

(9) $$A\left(\Delta T\right)=\frac{k_{1}k_{2}}{T_{p}}\int_{0}^{T_{p}}\left(x\left(t-T\right)x\left(t-T_{DL}\right)+y\left(t-T\right)y\left(t-T_{DL}\right)\right)dt.$$

The result of Integration (9) is a value independent of time *t*. However, this value (i.e., integration result) depends on the time difference *T* − *T_DL_*. When the value of *T_DL_* is set by the delay line and the value of time *T* is constant (constant position of the target), the output signal from correlator *S_OUT_* is also constant, and it reaches its maximum for *T* = *T_DL_*. Another situation takes place when a target is making micro-movement, for example, that described by the harmonic expression *D*(*t*) = *D* × cos(*ω*_0_ × *t*), where *D* is the amplitude of this movement. Then, the round-trip time *T* is also harmonically dependent on *t*. In effect, the output signal from the integrating circuit also depends on *t*. This means that the integrator output signal *S_OUT_* varies in time according to the varying distance from the radar to target and its frequency corresponds to the frequency of the target micro-movement.

There is the requirement that the micro-movement should be a slow-varying function of time compared to the integration time *Tp* of the integrating circuit. The value of micro-Doppler frequency must be lower than the cut-off frequency of the lowpass filter at the output of the correlator multiplying circuit.

Equation (8) describes the value of the correlation function for the noise signal transmitted and received by the noise radar [21,22,23,24].

## 3. New Concept of a Combined Microwave Delay Line with a Set of Fine Distance Gates

### 3.1. Combined Structure

In order to control the position of the detector operating point, an adjustable analogue delay system for micro-movement in a noise radar has to be used and it may be realized with the use of a combined delay line structure.

The two structures of microwave delay lines are as follows: the digitally-controlled cascaded line with switched delay sections and the analogue tapped delay line, which may be combined with one other. The resulting structure, formed by cascading, gains additional interesting features. The line with switched delay sections sets one time delay value from a predefined finite set, which may be called the coarse one. Furthermore, the delayed input signal enters the second line, which is the tapped delay line. The tapped line introduces several values of a smaller time delay for each tap output simultaneously. These delays may be called the fine ones and they are added to the coarse time delay set by the first line. As a result, there is a comb of time delays corresponding to radar range gates (spread by unit time delay of the tapped line), which is switched up and down by the value of unit time delay of the digitally-controlled first line (coarse step). The fine time delays offset should be sorted to evenly cover the unit time delay of the coarse line.

The combined line mentioned above does not ensure that the optimal operating point on the detector characteristic (with the meaning of point P1 on the autocorrelation function) corresponds to one of the fine gate delays. In order to find the optimal operating point for detection, the third delay line has to be cascaded. This additional delay line is the adjustable one, preferably in the form of an analogue precision phase shifter.

**Figure 2:**
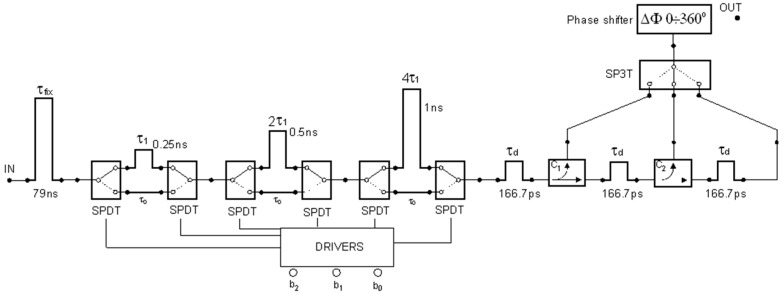
Example of a digitally-controlled delay line with a set of fine distance gates and an adjustable phase shifter for finding the optimal operating point of a correlation detector.

The innovative concept of such a combined line is shown in Figure 2, with an example of specified time delay implementation.

### 3.2. Analogue Tapped Cascaded Delay Line

Digitally-controlled microwave delay lines are considered here to realize a reference delay for a noise radar with analogue correlation. This type of radar performs analogue correlation of the received signal with a delayed version of the transmitted signal. However, compared to a radar with digital signal processing including signal cross-correlation, the analogue correlator only performs convolution for one selected value of time delay called the time gate.

In order to bring the functionality of a radar with an analogue correlator closer to the digital one, a special kind of delay line may be used, known as the analogue tapped delay line.

**Figure 3:**
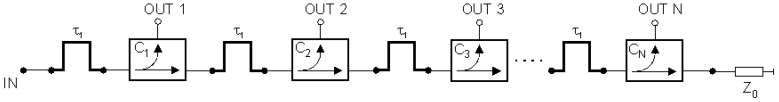
General scheme of an analogue tapped cascaded delay line.

In general, the analogue tapped delay line, known from the literature, consists of a number of unit delay sections and microwave couplers, having the same coupling factor, cascaded as shown in Figure 3 [25]. One unit delay line with one coupler forms one delay line stage. Every tap, which is a coupler’s coupled signal port, is the output of the delayed input signal, with a time delay equal to the unit time delay τ_1_ multiplied by the stage number.

According to this idea, every signal tap corresponds to one time gate and one signal correlator. The general scheme of an analogue tapped delay line is shown in Figure 3.

The application of this circuit causes a need to use several analogue correlator units equal to N, consisting of a signal mixer and a lowpass filter.

It is important to note that the analogue tapped delay line allows all signals corresponding to all time gates to be obtained quasi-simultaneously. Here, the term “quasi” means that the time gate output signals appear after subsequent unit time delay, but there is no need to use a signal switch (SPDT or SPST) to set the one desired value of time delay per one specified state of line. The analogue tapped cascaded delay line can be characterized by a number of features.

Advantages:

- Ability to obtain multiple values of the time delay quasi-simultaneously;
- Lack of unstable or transient states;
- Lack of microwave switches and their driving and biasing;
- Lower signal losses due to the absence of insertion losses of microwave switches.

Disadvantages:

- Need for high signal isolation between taps in order to minimize signal crosstalk;
- Maximal number of taps and therefore delay gates is a function of the signal coupling factor at each tap. In order to have a high number of taps, the coupling factor should be small, which results in the need for additional signal amplifying;
- Presence of insertion losses of microwave couplers;
- Different levels of output signal power at each tap appearing in descending order.

The design of a new concept of the analogue tapped delay line, which is proposed below, requires the design and optimization of dedicated microwave couplers with precisely chosen values of the coupling factor in order to obtain the same signal power level at each tap.

## 4. Numerical Validation

### 4.1. Optimization of the Correlation Detector Operating Point

**Figure 4:**
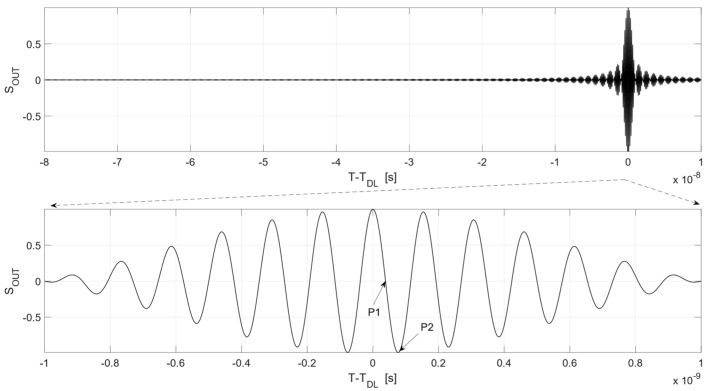
Plot of a normalized Function (8) for *T* = 80 ns, 6–7 GHz band, and *D*(*t*) = 0.

A normalized Function (8) for *T* = 80 ns, 6–7 GHz band, and *D*(*t*) = 0 is shown in Figure 4.

**Figure 5:**
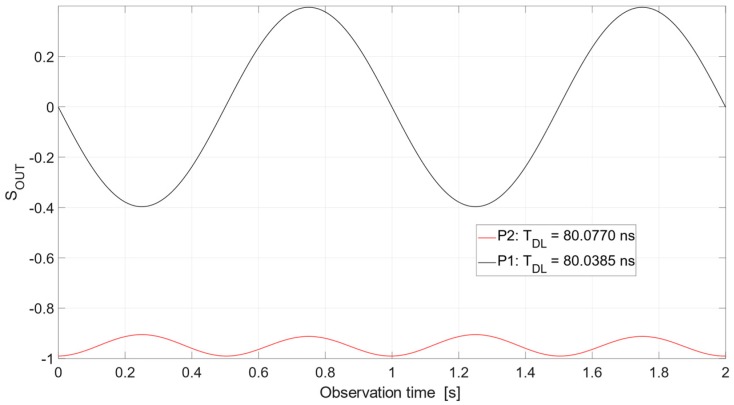
Illustration for the conversion of harmonic movement of an object to the output signal of the microwave correlation detector.

As can be seen in Figure 4, the noise radar may be applied for micro-movement detection. The operating points P1 and P2 of the correlation detector can be selected from the operational range shown in Figure 4. An example of the output signal of the correlation detector *S_OUT_*(*t*) for P1 and P2 is shown in Figure 5. The assumptions for this calculation are as follows: distance to object *R* = 12 m, harmonic micro-movement *D*(*t*) with amplitude 1 mm and frequency equal to 1 Hz, radar transmits noise signal with bandwidth *B* = 1 GHz, and center frequency *f*_0_ = 6.5 GHz. In this case, the point P1 corresponds to delay *T_DL_*_1_ and P2 corresponds to delay *T_DL_*_2_, which equal *T_DL_*_1_ = 80.0385 ns and *T_DL_*_2_ = 80.0770 ns. The use of a tunable microwave delay line is crucial in this case. The best operating point P1 for proper micro-movement detection is not placed for *T* = 80 ns, resulting from the distance between the radar and a target. Therefore, there is a need to correct the position of the operating point by introducing an additional value of time delay. It is possible to shift the operating point of the micro-movement detector due to the precise adjustment of time delay provided by the tunable delay line.

The operating point P1 is placed on the linear part of the detector characteristic and the detector output signal (black line in Figure 5) at this point properly corresponds to the shape and frequency of the micro-movement.

In contrast, the operating point P2 is placed on the nonlinear part of the detector characteristic and the detector output signal at this point (red line in Figure 5) incorrectly replicates the shape and frequency of the micro-movement. In particular, the micro-movement frequency is incorrect and equal to the doubled value of the proper frequency due to the even shape of the characteristic in the vicinity of the operating point.

### 4.2. Implementation of Time Delay Values

The example of the implementation of specified time delay values in a combined delay line structure, according to the proposed concept (Figure 2), assumes the case of micro-movement detection introduced above in Section 4.1.

The first line is the fixed delay line, which sets the time delay τ_fix_ corresponding to the distance 12 m lowered by the value of 1 ns, i.e., half of the autocorrelation function width (as shown in Figure 4). The digitally-controlled line with switched sections consists of three sections with the following time delay values: 0.25, 0.5, and 1 ns. It corresponds to a 3-bit control with unit time delay equal to 0.25 ns, and a maximal value of coarse time delay equal to 1.75 ns. Next, there is the tapped delay line with three taps and fine unit time delay equal to 166.7 ps. In effect, for every state of the digitally-controlled line, there are three values of fine gates shifted by 166.7 ps, which cover the range of 0.5 ns evenly.

The combined line presented in Figure 2 comprises one analogue phase shifter preceded by an SP3T switch. In this application, only one micro-Doppler detector is needed, and it is placed at the output of the phase shifter. This structure allows the optimal operating point for a correlation detector to be found in the case of a change of target position.

There is another variant of this solution that is possible when there are three correlation detectors connected to the subsequent output taps of the tapped line, and the phase shifter is placed between switched and tapped delay lines. In this case, there is no need for an SP3T switch (or in general, an N-way SPNT switch).

### 4.3. Calculations of Optimal Parameters of an Analogue Tapped Delay Line

According to the new concept, the main assumption for optimization of the tapped line design is that the same level of signal power is obtained at each tap, i.e., the coupled signal port.

Denoted in terms of power,

- Input power as *x*;
- Coupled signal power values as *y*_1_ to *y_N_*;
- Coupler’s direct output power values as *x*_1_ to *x_N_*;
- Power coupling factors *C*_1_ to *C_N_*;

where *N* is the maximal number of taps, for lossless couplers, one may notice

(10) $$x_{1}=\left(1-C_{1}\right)x$$

and

(11) $$y_{1}=C_{1}x.$$

Substituting further for *y*_2_ and *x*_2_ results in

(12) $$y_{2}=C_{2}x_{1}=C_{2}\left(1-C_{1}\right)x$$

and

(13) $$x_{1}=\left(1-C_{2}\right)x_{1}=\left(1-C_{1}\right)\left(1-C_{2}\right)x.$$

**Figure 6:**
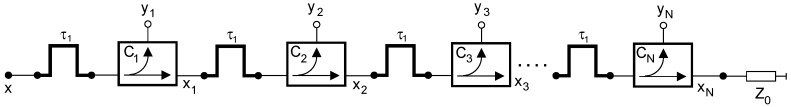
General scheme of a cascaded tapped delay line used in considerations, with variables denoted.

The considered situation is shown in Figure 6.

The condition for equal coupled signal powers is

(14) $$y_{1}=y_{2}=\ldots y_{N},$$

which gives the result

(15) $$C_{2}=\frac{C_{1}}{1-C_{1}}$$

and

(16) $$C_{N}=\frac{C_{N-1}}{1-C_{N-1}}.$$

Finally, by substituting coupling factors for subsequent taps, one may obtain

(17) $$C_{N}=\frac{C_{1}}{1-\left(n-1\right)C_{1}}.$$

Because the last tap does need not a coupler, the value of *C_N_* equals 1 and using Equation (17), one may find

(18) $$C_{N}=\frac{1}{N+1-n}.$$

The equation above (18) expresses the value of the power coupling factor as a function of the tap number *n* for the assumed overall number *N* of taps. With the use of this equation, the example values of coupling factors were calculated and are presented in Table 1. The calculations were done for three values of the overall tap number, i.e., *N* = 4, 8, and 16.

When the design guidelines above are implemented, the power transmission factor for every tap output with respect to the main input of the whole delay line is the same and equals 1/*N*. This is true for lossless unit delay lines and couplers; when these devices are lossy, the insertion losses accumulate and increase with the tap number.

Assuming that the constant tapped power rule is taken into account, the design methodology of the microwave tapped analogue delay line should follow

- Choice of unit time delay value;
- Choice of maximum number of taps (and unit delays) for the whole delay structure;
- Calculation of power coupling factors for subsequent taps *C*_1_ to *C_N_*;
- Choice of technology for physical realization of the unit delay line and couplers;
- Dedicated design or purchase of the unit delay line and set of couplers with specified, previously calculated, coupling factors.

Further optimization of the whole delay line structure, e.g., possible integration of coupling structures with circuits of unit delay lines, can be pursued in the case of one’s own design (especially possible when planar technology is chosen).

## 5. Discussion

For very high frequencies in the microwave range, the simple analogue systems of the correlative detector with a controlled microwave delay line can be used, because digital realization of the micro-movement detector for microwave frequencies is very difficult and demands very high-speed analogue-to-digital conversion. As far as existing delay line technologies are concerned, the conclusion is that, in spite of constant development, there is no ideal solution, which would join the features like low losses, a high delay, a low cost, and small dimensions.

The choice of delay line solution depends on the expected application. One may use commercially available components or design one’s own dedicated solutions. When the type and technology of unit delay lines is chosen, the whole structure of the delay line set should be considered in order to fulfill the requirements of the given project.

For the radar (especially noise one) with an analogue correlator, the optimal solution seems to be a combined delay line consisting of switched and tapped parts. The parameters like the number of control bits, number of delay states, and unit delay value are matched in order to optimize signal losses, bandwidth, structure complexity, maximal detection range, and range gate grid.

There are various possible applications of combined delay lines when noise radar is used:

1. Surveillance of an area defined by a set of subsequent range gates in order to detect the entrance of an object in the monitored area. Then, the combined delay line, which consists of a digitally-switched delay line and a cascaded tapped delay line without an SPNT switch and phase shifter(s), is a sufficient solution;
2. Solution as above assuming that the surveillance range is fixed. Then, there is no need for switching, the set of delay lines mentioned above can be simplified to one fixed delay line cascaded by a tapped delay line with the number of taps equal to the number of desired additional monitoring range gates. This solution may be especially suitable for systems designed to protect an object (vehicle, building) against an attack, for example, protection of a tank against Rocket-Propelled Grenades;
3. Monitoring the vital signs of a stationary person, for example, a patient in bed or a worker at the desk. In these cases, when the distance to the person in known, the fixed delay line may be used, followed by a tapped delay line with several delay stages. The tapped line delays cover the predicted delay value corresponding to the width of the autocorrelation function. The outputs of the tapped line may be connected to the SPNT switch and adjustable phase shifter or to a corresponding number of phase shifters directly.

The natural competition for analogue signal processing methods in a noise radar are digital techniques, which rely on calculation of the correlation function in the digital domain. This solution requires direct analog-to-digital (AD) conversion of the microwave signal in a given bandwidth. However, for very high frequencies in the microwave range, simple analogue systems of the correlative detector can still be used, because direct sampling of the microwave signal and digital realization of the autocorrelation function for these frequencies are very difficult and require extremely fast AD conversion.

The methods presented in this paper use an analogue correlation detection in the microwave band, in contrast to processing of the received signal in the primary band. This allows a movement using an internal structure of the correlation function of a noise signal to be precisely detected. The solution that uses the digitally-controlled switched delay line cascaded with the tapped delay line allows the features of synthesizing the big delay values (switched line) to be combined with simultaneously obtaining several values of the time delay (tapped line). An additional precision analogue phase shifter allows the optimal operating point of a micro-Doppler detector to be found.

## Figures and Tables

**Table 1:** Calculated values of power coupling factors for values of the overall tap number: *N* = 4, 8, and 16.

*N* = 4	*N* = 8	*N* = 16	*C_n_*	*C_n_* (dB)
-	-	C_1_	1/16	−12.04
-	-	C_2_	1/15	−11.76
-	-	C_3_	1/14	−11.46
-	-	C_4_	1/13	−11.14
-	-	C_5_	1/12	−10.79
-	-	C_6_	1/11	−10.41
-	-	C_7_	1/10	−10
-	-	C_8_	1/9	−9.54
-	C_1_	C_9_	1/8	−9.03
-	C_2_	C_10_	1/7	−8.45
-	C_3_	C_11_	1/6	−7.78
-	C_4_	C_12_	1/5	−6.99
C_1_	C_5_	C_13_	1/4	−6.02
C_2_	C_6_	C_14_	1/3	−4.77
C_3_	C_7_	C_15_	1/2	−3
C_4_	C_8_	C_16_	1	0
